# Health, work and demographic factors associated with a lower risk of work disability and unemployment in employees with lower back, neck and shoulder pain

**DOI:** 10.1186/s12891-019-2999-9

**Published:** 2019-12-26

**Authors:** Lisa Mather, Annina Ropponen, Ellenor Mittendorfer-Rutz, Jurgita Narusyte, Pia Svedberg

**Affiliations:** 1 Division of Insurance Medicine, Department of Clinical Neuroscience, Karolinska Institutet, Stockholm, Sweden; 2 Finnish Institute of Occupational Health, Helsinki, Finland

**Keywords:** Back pain, Sick leave, Unemployment, Work disability, Disability pension, Psychosocial work environment, Self-rated health, Physical activity, Common mental disorders

## Abstract

**Background:**

Chronic musculoskeletal pain affects over 20% of the adult population and is one of the most common reasons for sick leave in Sweden. The aim of this study was to investigate which demographic, health and psychosocial work environment factors are of importance for a lower risk of future work disability and unemployment among workers with low back pain (LBP) and/or neck shoulder pain (NSP), and if familial factors influence these associations.

**Methods:**

All 5556 persons that reported having LBP and/or NSP in a web-based questionnaire study in 2004–2006 were included. They were followed up for work disability (sick leave > 90 days or disability pension), and unemployment (> 180 days in a year) until 31 December 2013. Hazard ratios (HR) with 95% confidence intervals were calculated using cox proportional hazard models of the whole sample, adjusting for covariates. In addition, co-twin analyses of outcome discordant twin pairs were conducted to assess the impact of familial confounding on the associations.

**Results:**

Being male, 19–28 years old, having higher education, only NSP, no history of depression or anxiety, good self-rated health, low job demands and high job control were associated with a lower risk of work disability (adjusted HR ranging between 0.29–0.85). No history of anxiety and depression and high job control was associated with a lower risk of unemployment (adjusted HR ranging from 0.53 and 0.67). Familial factors were found to affect the association between education and work disability, but none of the other associations investigated.

**Conclusions:**

Among those with LBP or NSP, good health in terms of mental- and self-rated health, few pain sites, as well as good psychosocial working conditions seem to indicate a lower risk for work disability.

## Background

Chronic musculoskeletal pain, that affects over 20% of the adult population, is the most common cause of severe, long-term, physical disability [1]. In Sweden it is estimated that 5% of the population have back pain that prevents them from their normal daily activities [2] and back pain is one of the most common reasons for sick leave [3]. Moreover, unemployment rates are usually twice as high for people with health problems or disability such as chronic pain [4]. Encouraging work participation in people with these types of disabilities is an important issue, both for the economy and to avoid marginalization [4]. Knowing the factors that are associated with a lower risk of work disability and unemployment for those with chronic pain may be helpful for future studies and interventions.

Among those with back pain, psychosocial and work-related factors have been shown to affect the risk of sick leave and disability pension [5]. A study of Danish workers with low back pain (LBP) and neck and/or shoulder pain (NSP), found that pain intensity and heavy physical work increased the risk of sick leave [6]. Moreover, two recent studies showed that the number of pain sites independently predicted sick leave and disability pension among those with back pain [7, 8]. Having comorbidity of back pain and common mental disorders, such as depression and anxiety, increases the risk of disability pension 15–20 fold [9]. This is a cause of concern as in Europe, up to one third of persons with chronic pain conditions, most frequently back pain, have been diagnosed also with depression [10, 11]. Even though less studied, previous research has also pointed out the importance of considering employment as an outcome when studying back pain, and that other factors than pain related seem to influence the increased risk of unemployment among those with back pain such as depression [12, 13].

Back pain is more common among women than men, among blue collar than white collar workers, and the prevalence increases with age [2]. Risk factors include both physical, psychological and genetic factors [14], as well as a poor psychosocial work environment with high demands and low control [15]. Previous studies have also found that physical activity reduce disability associated with back pain [16]. A twin study found that additive genetic factors explained 24–30% of the variance of the separate pain sites and 60% of the variance for concurrent NSP and LBP [17]. Further, since sick leave and disability pension have been found to be moderately heritable [18–20]. Hence, there is a possibility that familial factors (genes and shared environment) would affect the associations. Studying twins that share 50–100% of their genetic material and 100% rearing environment (when raised together) provides a possibility to adjust for those unmeasured factors, which are hereafter referred to as familial factors. With a co-twin control design (matched case-control analysis) an exposure is evaluated after controlling for genetic predisposition and environment, while growing up. This is then compared to results of an analysis of the whole sample, where the twins are treated as individuals to assess the influence of familial factors, as is a common approach in twin studies [21, 22]. By taking familial factors into account more accurate estimates of risk factors for work disability may be provided, which supplement the epidemiological findings of unrelated subjects. Few previous studies have investigated factors protective against work disability taking familial factors into account.

The aim of this study was to investigate what demographic, health and psychosocial work environment factors, are of importance for a lower risk of future work disability and unemployment among workers with LBP or NSP, and if familial factors influence these associations.

## Materials and methods

### Study population

The study population was based on the Swedish Twin project Of Disability pension and Sickness absence (STODS). Data from the Study of Twin Adults Genes and Environment (STAGE), a web-based questionnaire study performed by the Swedish Twin Registry (STR) in 2004–2006, are included as well as national register data. STAGE was sent to all twins in the STR born between 1959 and 1985 (19–47 years old at baseline in 2004–2006 and 28–58 years old in 2013) and contains responses from 25,496 twins (60% response rate) [23, 24]. For this study, the respondents with LBP and/or NSP during the past 6 months were included.

When assessing back pain, it is important to take site, severity and pain related disability into account, in order to achieve valid standardized measures [25]. LBP and NSP are measured using an index that covers both pain intensity and pain-related disability [17]. NSP and LBP are separate conditions but comorbidity is common [14, 17]. Hence, LBP and NSP were assessed as per Nyman and colleagues [17]. The questions were based on the Standardized Nordic questionnaires for the analysis of musculoskeletal symptoms [26], that has been shown to have good to excellent reliability [27]. The initial question was: “Have you during the past 6 months had pain, ache, discomfort somewhere in your body?” The answer involved marking a drawing of where the pain was located. Individuals that marked the shoulders, neck, or lower back was asked 3 additional questions on pain intensity and 3 questions on pain-related disability. If a pain intensity score 3 or more and/or a pain-related disability score 1 or more was present in the neck or shoulders, the person was classified as having NSP and if it was present in the lower back as having LBP [17]. Three categories of pain locations in i.e. LBP, NSP, or LBP and NSP were included [17].

**Fig. 1:**
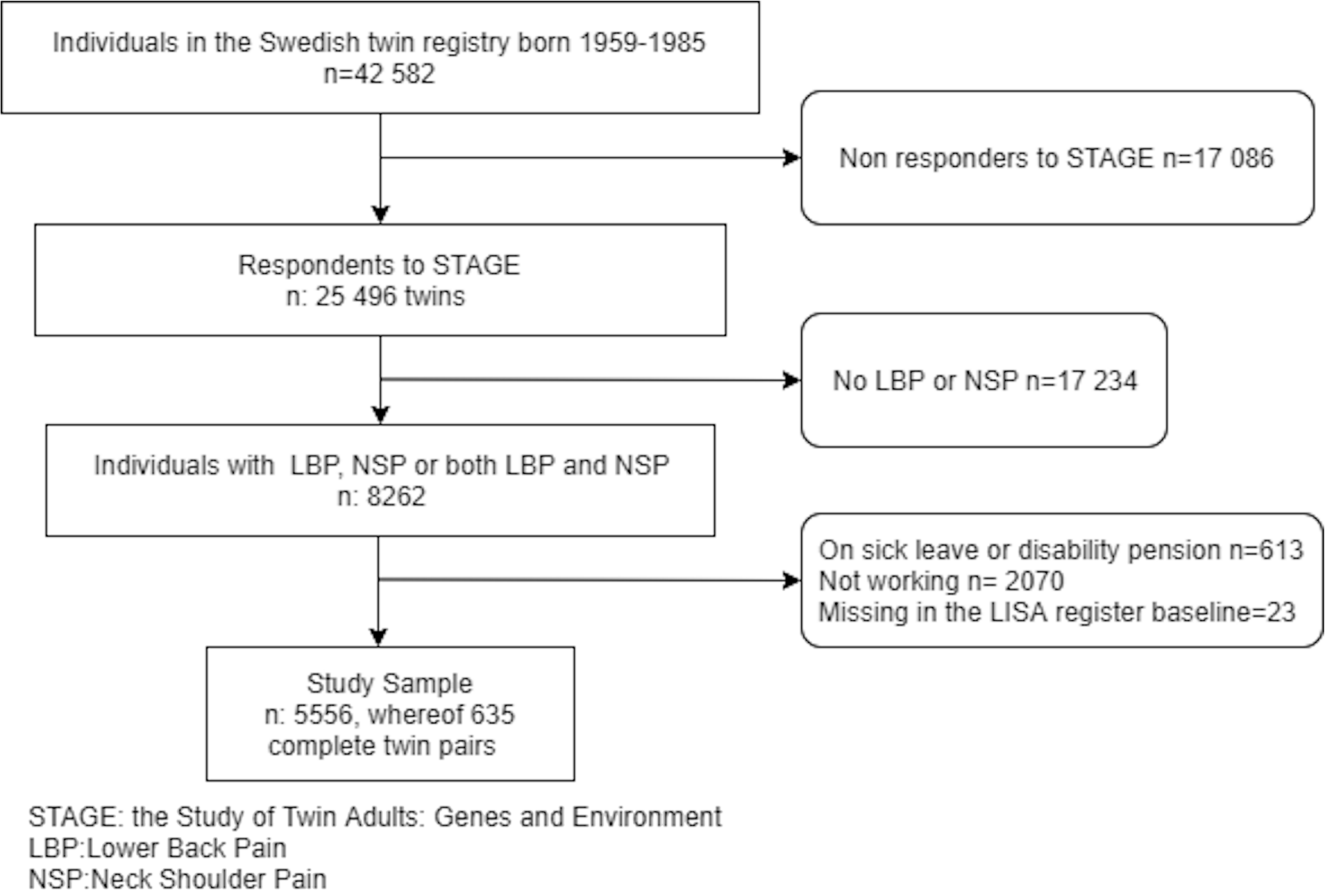
Flow chart of the study sample

Inclusion criteria were no sick leave or disability pension at baseline (response date to STAGE) or previously granted disability pension. Moreover, only those that reported to be working and who were present in the Longitudinal Integration Database for Health Insurance and Labour Market Studies (LISA) [28] at baseline were included (Fig. 1). Twin individuals were included in the study even if their co-twin did not respond or meet inclusion criteria, since they can still contribute to analyses of the whole sample. In the final sample of 5556 twin individuals (Table 1), there were 635 complete twin pairs whereof 281 monozygotic, 174 dizygotic same sex, 10 of unknown zygosity, and 170 opposite sex. The sample also contains 4286 single twins where the co-twin did not meet inclusion criteria.

**Table 1:** Frequencies of demographic, work and health factors in the 5556 Swedish individual twins with Lower Back Pain (LBP), Neck and/or Shoulder Pain (NSP), stratified on sex

	All (*N* = 5556)	Men (*n* = 2193)	Women (*n* = 3363)
	n (%)	n (%)	n (%)
*Age group*			
19–28	905 (16.3)	339 (15.5)	566 (16.8)
29–37	1946 (35.0)	765 (34.9)	1181 (35.1)
38–47	2705 (48.7)	1089 (49.7)	1616 (48.1)
*Education*			
Primary	370 (6.7)	183 (8.3)	187 (5.6)
Secondary	2557 (46.0)	1138 (51.9)	1419 (42.2)
Higher	2245 (40.4)	774 (35.3)	1471 (43.7)
Missing	384 (6.9)	98 (4.5)	286 (8.5)
*Back pain*			
LBP	1576 (28.4)	821 (37.4)	755 (22.5)
NSP	2518 (45.3)	872 (39.8)	1646 (48.9)
LBP & NSP	1462 (26.3)	500 (22.8)	962 (28.6)
*MDD/GAD*			
No	3940 (70.9)	1740 (79.3)	2200 (65.4)
Yes	661 (11.9)	165 (7.5)	496 (14.7)
Missing	955 (17.2)	288 (13.1)	667 (19.8)
*Self-rated health*			
Excellent	1241 (22.3)	540 (24.6)	701 (20.8)
Good	2856 (51.4)	1136 (51.8)	1720 (51.1)
Moderate	1235 (22.2)	445 (20.3)	790 (23.5)
Not good/Poor	222 (4.0)	72 (3.3)	150 (4.5)
Missing	2 (0)	0 (0)	2 (0.1)
*Physical activity*			
None/low	921 (16.6)	346 (15.8)	575 (17.1)
Moderate	1262 (22.7)	402 (18.3)	860 (25.6)
High	1529 (27.5)	533 (24.3)	996 (29.6)
Vigourous	773 (13.9)	333 (15.2)	440 (13.1)
Missing	1071 (19.3)	579 (26.4)	492 (14.6)
*Regular analgesia*			
No	3243 (58.4)	1550 (70.7)	1693 (50.3)
Yes	2309 (41.6)	642 (29.3)	1667 (49.6)
Missing	4 (0.1)	1 (0)	3 (0.1)
Mean job demands	2.4 (SD 0.6)	2.4 (SD 0.6)	2.4 (SD 0.6)
Mean job control	2.0 (SD 0.6)	1.9 (SD 0.6)	2.0 (SD 0.6)
Mean job Support	1.7 (SD 0.5)	1.7 (SD 0.5)	1.7 (SD 0.5)
*Work environment*			
Low strain	1223 (22.0)	506 (23.1)	717 (21.3)
Passive	900 (16.2)	332 (15.1)	568 (16.9)
Active	1891 (34.0)	819 (37.3)	1072 (31.9)
Strain	438 (7.9)	155 (7.1)	283 (8.4)
Iso-strain	698 (12.6)	230 (10.5)	468 (13.9)
Missing	406 (7.3)	151 (6.9)	255 (7.6)
*Sick leave > 90 net days or disability pension*			
Yes	761 (13.7)	207 (9.4)	554 (16.5)
No	4795 (86.3)	1986 (90.6)	2809 (83.5)
*Unemployment > 90 days*			
No	4954 (89.2)	1978 (90.2)	2976 (88.5)
Yes	602 (10.8)	215 (9.8)	387 (11.5)

### Outcomes

*Work disability* was defined as Sick leave > 90 net days or disability pension incidence assessed in the MicroData for Analysis of the Social Insurance database (MiDAS) register held by the Social Insurance Agency that contains dates, duration and grade of sick leave and disability pension [29]. The follow-up time was from the STAGE response date until 31 December 2013. Sick leave and disability pension can be granted 25, 50, 75% or 100% of regular working hours and the net days are calculated taking part time sick leave into account, i.e. two half sick leave days is calculated as 1 net day. For disability pension all incident cases were included. There were 140 twin pairs discordant for work disability (280 individuals).

*Unemployment > 90 days* in a calendar year was assessed in LISA held by Statistics Sweden [28]. LISA contains demographic, and work-related information on all residents of Sweden including amount of days being registered with the Swedish public employment service as a job seeker. Follow-up was from the year after STAGE response until 31 December 2012. Since we only had the amount of days per year available from LISA, the follow up date was set as 1 January the year the unemployment occurred. There were 90 twin pairs discordant for unemployment (180 individuals).

### Exposures

#### Demographics

*Sex* was used as a dichotomous variable man/women and women were set as the reference category. *Age group* was divided into 19–28, 29–37 and 38–47 years (at baseline) and the oldest group was the reference category. *Education* was self-reported and classified as primary education (9 years), secondary education (12 years), and higher education (> 12 years). Secondary education was used as reference category.

#### Health

*Self-rated health* (SRH) was measured with the question “How would you rate your general health status?” with the responses excellent, good, moderate, fairly poor or poor. Poor and fairly poor had few respondents so the categories were collapsed and used as the reference category. *Physical activity* was obtained from a question asking the participants to rate their physical activity from 1 to 10 and the variable was coded as: none (1–2), low (3–4), moderate (5–6), high (7–8), and vigorous (9–10) [30]. None and low were collapsed due to few respondents and moderate was used as the reference category. *Analgesic use* was obtained from a question inquiring about the regular use of Over The Counter (OTC) analgesics and was coded as 0 = no and 1 = yes. Regular use was used as the reference category. Comorbidity with common mental disorders i.e., lifetime prevalence of Major Depressive Disorder (MDD) and/or Generalized Anxiety Disorder (GAD) were assessed with 38 and 23 questions respectively, based on the Diagnostic and Statistical Manual of Mental Disorders, fourth edition (DSM-IV) criteria for each disorder and the Structured Clinical Interview for DSM-IV Disorders [31]. Criteria A, C and E had to be present for the participant to be classified as having had MDD and criteria A and C had to be present in order for the participants to be classified as having had GAD [32]. The variable was coded as 1 = the presence of MDD or GAD and 0 = no MDD or GAD.

#### Psychosocial work environment

*Job Demands, Control and Support* was based on the Swedish version of Karasek and Theorell’s measure [33]. Demands measure psychological demands at work with questions such as: “Does your job require too great a work effort?”, control measures decision latitude with questions such as: “Do you have the possibility to decide for yourself how to carry out your work?” and support measures social support at work with questions such as: “There is good collegiality at work”. The measure was used both as separate continuous variables, and using median as a cutoff and a categorical variable was created. High demands/high control was an active job. Low demands and low control a passive job. Low demands and high control was low strain. High demands and low control was job strain and high demands, low control and low support was iso-strain. Iso-strain was used as the reference category.

### Censoring variables

*Emigration* was measured by whether an individual was missing in the LISA register for two consecutive years (not living in Sweden), the 1 January the first missing year was used as censoring date. *Date of death* was assessed in the cause of death register, held by the National Board of Health and Welfare. The analyses were also censored for the respective outcomes and end of follow-up.

### Statistical analyses

Cox proportional hazards regression models were performed to assess Hazard Ratios (HR) with 95% Confidence Intervals (CI) in STATA IC12, using the clustered robust standard error to take into account that the sample contained twins. *P*-values < 0.05 were considered statistically significant. Variables were first analyzed individually, and in the second step adjusted for sex, age and education. In the third step all variables that showed a significant association with the outcome were added into the same model. Conditional Cox regression analyses were also performed on the discordant twin pairs, where one twin had the outcome and the other did not. This analysis control for factors shared by the co-twins i.e. approximately 50% of genetics for dizygotic twins and 100% of genetics for monozygotic twins, as well as shared environment for both types of twins, i.e. familial factors. This analysis is then compared to the analyses of the whole sample to assess the influence of familial factors on the associations. If the HRs in the co-twin model are lower than that of the whole sample, we can suspect that familial factors are of importance in the associations [21, 22].

## Results

The majority of the sample were women (60.5%), approximately half of the sample was 38–47 years old (48.7%), and having only NSP (45.3%) was most common (Table 1). We found that 13.7% had incident sick leave (> 90 days) or disability pension and 10.8% were unemployed for more than 90 days in a year during follow-up (Table 1). Both sick leave/disability pension and unemployment were more common among women compared to men (*p* < 0.001 and 0.046).

### Demographics

Women had a higher risk of sick leave/disability pension compared to men (Table 2), however, for unemployment there were no significant sex difference (Table 3).

**Table 2:** Hazard ratios for sick leave> 90 days/disability pension with 95% confidence intervals

	Crude	Adjusted sex, age and education	Adjusted for all significant variables	Co-twin analyses of discordant twin pairs (*n* = 140 pairs)
*Sex*				
Male	**0.56 (0.47–0.65)**		**0.57 (0.47–0.69)**	
Female	ref		ref	
*Age*				
19–28	**0.62 (0.49–0.78)**		**0.53 (0.41–0.69)**	
29–37	**0.85 (0.73–0.99)**		0.84 (0.71–1.01)	
38–47	ref		ref	
*Education*				
Primary	**1.31 (1.02–1.70)**		1.20 (0.91–1.58)	0.84 (0.33–2.13)
Secondary	ref		ref	ref
Higher education	**0.75 (0.64–0.88)**		**0.75 (0.63–0.89)**	1.06 (0.55–2.05)
*Back pain*				
LBP	**0.60 (0.49–0.72)**	**0.70 (0.57–0.86)**	0.88 (0.71–1.09)	0.60 (0.32–1.12)
NSP	**0.71 (0.61–0.84)**	**0.70 (0.59–0.83)**	**0.79 (0.66–0.95)**	0.81 (0.48–1.34)
LBP & NSP	ref	ref	ref	ref
*MDD/GAD*				
No	**0.48 (0.41–0.56)**	**0.49 (0.42–0.58)**	**0.58 (0.48–0.69)**	**0.44 (0.24–0.81)**
Yes	ref	ref	ref	ref
*Self-rated health*				
Excellent	**0.21 (0.16–0.29)**	**0.23 (0.17–0.31)**	**0.30 (0.22–0.43)**	**0.30 (0.10–0.87)**
Good	**0.28 (0.22–0.37)**	**0.30 (0.23–0.39)**	**0.38 (0.29–0.51)**	0.44 (0.18–1.09)
Moderate	**0.50 (0.38–0.64)**	**0.50 (0.38–0.65)**	**0.56 (0.42–0.75)**	0.65 (0.26–1.59)
Not good/Poor	ref	ref	ref	ref
*Health behaviours*				
Physical activity				
None/low	1.09 (0.87–1.36)	1.12 (0.89–1.41)		1.40 (0.73–2.71)
Moderate	ref	ref		ref
High	0.97 (0.79–1.18)	1.00 (0.81–1.23)		1.00 (0.52–1.94)
Vigourous	**0.72 (0.56–0.94)**	0.80 (0.60–1.05)		0.50 (0.19–1.27)
*Regular analgesia*				
No	**0.76 (0.66–0.88)**	**0.82 (0.71–0.96)**	0.89 (0.76–1.05)	**0.60 (0.37–0.98)**
Yes	ref	ref	ref	
Missing				
*Work variables*				
Job demands 1–4				
First 4 years	**0.69 (0.58–0.81)**	**0.69 (0.58–0.83)**	**0.79 (0.66–0.95)**	0.86 (0.49–1.50)
After 4 years	0.93 (0.77–1.12)	0.90 (0.74–1.08)	0.96 (0.78–1.17)	1.12 (0.56–2.23)
Job control 1–4	**0.82 (0.73–0.92)**	**0.82 (0.72–0.94)**	**0.85 (0.73–0.98)**	0.83 (0.53–1.28)
Job Support 1–4				
First 4 years	**0.72 (0.60–0.86)**	**0.74 (0.61–0.89)**	1.00 (0.82–1.23)	0.76 (0.41–1.38)
After 4 years	1.01 (0.79–1.28)	0.97 (0.76–1.24)	1.22 (0.94–1.60)	1.06 (0.47–2.36)
Low strain	**0.64 (0.50–0.82)**	**0.62 (0.48–0.80)**	0.79 (0.61–1.03)	1.02 (0.45–2.30)
Passive	**0.76 (0.59–0.98)**	**0.75 (0.58–0.98)**	0.88 (0.68–1.16)	1.07 (0.45–2.54)
Active	**0.78 (0.62–0.96)**	0.81 (0.64–1.02)	0.94 (0.74–1.19)	0.89 (0.43–1.82)
Strain	0.88 (0.65–1.18)	0.83 (0.61–1.13)	0.98 (0.71–1.35)	0.84 (0.32–2.21)
Iso-strain	ref	ref	ref	ref

Statistically significant HRs are marked in bold

**Table 3:** Hazard ratios for unemployment with 95% confidence intervals

	Crude	Adjusted sex, age and education	All significant variables in same model	Co-twin analyses of discordant twin pairs (*n* = 93 pairs)
*Sex*				
Male	0.84 (0.71–1.00)			
Female	ref			
*Age*				
19–28	**1.57 (1.27–1.94)**		**1.38 (1.09–1.74)**	
29–37	1.14 (0.95–1.38)		1.09 (0.89–1.35)	
38–47	ref		ref	
*Education*				
Primary	**1.90 (1.45–2.48)**		**1.91 (1.44–2.52)**	2.55 (0.76–8.59)
Secondary	ref		ref	
Higher education	0.87 (0.73–1.05)		0.95 (0.78–1.16)	1.05 (0.46–2.39)
*Back pain*				
LBP	**0.68 (0.55–0.85)**	**0.74 (0.59–0.93)**	0.80 (0.63–1.02)	**0.38 (0.17–0.87)**
NSP	0.83 (0.69–1.00)	0.85 (0.70–1.04)	0.90 (0.73–1.11)	0.92 (0.50–1.72)
LBP & NSP	ref	ref	ref	ref
*MDD/GAD*				
No	**0.59 (0.47–0.73)**	**0.62 (0.49–0.78)**	**0.68 (0.55–0.83)**	0.55 (0.27–1.10)
Yes	ref	ref	ref	ref
*Self-rated health*				
Excellent	**0.61 (0.42–0.88)**	**0.66 (0.45–0.97)**	0.88 (0.58–1.34)	0.96 (0.22–4.25)
Good	**0.56 (0.40–0.80)**	**0.60 (0.41–0.86)**	0.76 (0.51–1.13)	1.38 (0.35–5.44)
Moderate	0.91 (0.63–1.30)	0.91 (0.62–1.32)	1.01 (0.67–1.50)	1.24 (0.33–4.74)
Not good/Poor	ref	ref	ref	ref
*Physical activity*				
None/low	1.09 (0.84–1.41)	1.08 (0.82–1.41)		0.72 (0.31–1.69)
Moderate	ref	ref		ref
High	1.08 (0.86–1.36)	1.06 (0.83–1.35)		1.17 (0.53–2.55)
Vigourous	0.85 (0.63–1.14)	0.91 (0.67–1.23)		0.84 (0.31–2.25)
*Regular analgesia*				
No	0.96 (0.82–1.13)	0.99 (0.83–1.18)		0.82 (0.47–1.43)
Yes	ref	ref		ref
*Work variables*				
Job demands 1–4	0.95 (0.81–1.10)	0.91 (0.77–1.07)		0.87 (0.53–1.44)
Job control 1–4	**0.55 (0.48–0.63)**	**0.60 (0.52–0.69)**	**0.62 (0.52–0.72)**	0.59 (0.34–1.02)
Job support 1–4	**0.70 (0.59–0.84)**	**0.69 (0.57–0.82)**	0.85 (0.70–1.03)	0.88 (0.53–1.45)
Low strain	**0.50 (0.38–0.66)**	**0.52 (0.39–0.69)**	**0.58 (0.43–0.77)**	0.47 (0.18–1.22)
Passive	**0.90 (0.70–1.17)**	0.87 (0.66–1.14)	0.95 (0.72–1.25)	0.95 (0.32–2.87)
Active	**0.56 (0.44–0.71)**	**0.61 (0.47–0.79)**	**0.64 (0.49–0.84)**	0.68 (0.28–1.67)
Strain	0.71 (0.51–1.00)	**0.68 (0.48–0.97)**	0.75 (0.53–1.06)	0.91 (0.30–2.72)
Iso-strain	**ref**	**ref**	ref	ref

Statistically significant HRs are marked in bold

The youngest age group had lower risk of sick leave/disability pension compared to the oldest age group (Table 2), however, the youngest age group had a higher risk of unemployment compared to the oldest age group (Table 3).

Those with a higher education had a lower risk of sick leave/disability pension compared to those with secondary education in the analysis of the whole sample. However, the association seem to be influenced by familial factors as the HRs were above one and non-significant in the co-twin analysis (Table 2). This result indicates that the association was explained by genetics and shared environment. In the analysis for the risk of unemployment, having only a compulsory education was a risk factor but there was no significant difference between secondary or higher education. These associations do not seem to be influenced by familial factors (Table 3).

### Health

Having only one pain location compared to two was associated with a lower risk of both sick leave/disability pension and unemployment. However, the results did not reach statistical significance in the fully adjusted model except for NSP as a protective factor for sick leave/disability pension. The HRs pointed in the same direction in the co-twin analyses with indication of no major influence of familial factors for work disability (Table 2). However, in the co-twin analysis for unemployment, there was a decrease in the HR for LBP, indicating a possible interaction between familial factors and LBP on unemployment (Table 3).

Never having had MDD or GAD was associated with a lower risk of sick leave/disability pension and unemployment and these associations does not seem to be influenced by familial factors as the HRs were similar in the co-twin model (Tables 2 and 3).

Having excellent, good or moderate SRH was associated with a lower risk of sick leave/disability pension and this association does not seem to be influenced by familial factors (Table 2). However, in the analyses with unemployment as an outcome it did not reach statistical significance in the full model and the association seem to be influenced by familial factors (Table 3).

Vigorous physical activity was associated with a lower risk of sick leave/disability pension in the crude analyses, but this association was not statistically significant in the adjusted model (Table 2). There were no statistically significant associations between physical activity and unemployment (Table 3).

Not taking Over The Counter (OTC) analgesics were associated with a lower risk of sick leave/disability pension, but the association was not significant in the fully adjusted model (Table 2). There was no association between the use of OTC analgesics and unemployment (Table 3).

### Psychosocial work environment

Low job demands, high job control and high job support were all associated with a lower risk of sick leave/disability pension in the crude analysis and low job demands and high control remained significantly associated in the adjusted analysis (Table 2). Job control and support were associated with unemployment in the crude analysis, but only control remained significantly associated in the fully adjusted analysis (Table 3). There seemed to be no major influences by familial factors on the associations between psychosocial work environment and the outcomes.

## Discussion

This prospective study investigated which demographic, health and psychosocial work environment factors are of importance for a lower risk of future work disability and unemployment among young and middle aged workers with LBP and/or NSP. We found that being male, 19–28 years old, having higher education, only one pain location (NSP), no history of depression or anxiety, good SRH, low job demands and high job control were associated with a lower risk of sick leave/disability pension. No history of anxiety and depression and high job control were associated with a lower risk of unemployment. Familial factors were found to play a role in some of the associations, they explained the association between education and work disability, while adjusting for familial factors made the association between LBP and unemployment stronger. This is in line with previous research that has found both back pain and work disability heritable [17–20]. These findings also indicate that the heritability of back pain and work disability is shared with other factors.

### Demographics

Our finding that men had lower risk of sick leave/disability pension than women are in line with findings of the whole Swedish population [3]. The 19–28-year-old age group had a lower risk of sick leave, which is also in line with national statistics [34]. However, the younger age group had a higher risk of unemployment compared to the oldest age group. This may be since older workers are more established in the job market and more aware of their right to sick leave benefits, while young workers more often are on temporary contracts [35] and may expect not get the contract extended due to health problems, but instead end up unemployed. The fact that higher education seems protective for sick leave/disability pension has also been found in other studies [36, 37]. Our result that familial factors seem to be of importance in the association between education and sick leave is also in line with one previous twin study [38].

### Health

Those having only NSP had a lower risk of sick leave/disability pension compared to those with both LBP and NSP and no association was found for only LBP. The fact that multiple pain sites indicate higher risk of sick leave/disability pension is in line with previous studies [7, 8]. Never having had depression and/or anxiety was protective against both sick leave/disability pension and unemployment and familial factors did not influence the associations. Having excellent, good or moderate SRH was associated with a lower risk of sick leave/disability pension however the association did not reach statistical significance in the analyses with unemployment as an outcome. Previous research has found that mental disorders among young adults increase not only the risk of sick leave but also unemployment [39]. Moreover, pain and common mental disorders including depression and anxiety has been found to have a synergistic effect on disability pension and having both conditions increase the risk 19 fold for men and 15 fold for women [6].

Physical activity and regular use of over the counter analgesics did not show an independent association with sick leave/disability pension. Perhaps due to that they reflect the severity of the LBP/NSP rather than independent risk factors.

### Psychosocial work environment

Job demands and control were independently associated with sick leave/disability pension, however job control only was associated with unemployment in the adjusted analysis. The fact that psychosocial work environment are associated with sick leave has been found in previous studies of the general population [40, 41], as well as in twin studies [42, 43]. Contrary to expectations few of the associations were influenced by familial factors [42, 43].

### Strengths and limitations

Strengths of this study include the large population-based cohort of twins. Moreover, the use of register data minimizes loss to follow up and eliminates recall bias. Further strengths include the use of objective sick leave, disability pension and unemployment data of high quality with complete coverage, the prospective cohort design, and extensive survey data including validated measures of LBP and NSP and relevant confounders. A unique strength includes the possibility to control for familial confounding using the discordant twin pairs. Weaknesses include the low power in the co-twin analysis, making it difficult to draw firm conclusions on the effects of familial confounding, this low power also meant we could not analyze monozygotic and dizygotic twins separately, which would have given an indication of if it was genetic or shared environmental factors that was responsible for the familial confounding. Moreover, LBP and NSP includes a variety of back problems and previous research has found that the length of sick leave for musculoskeletal disorders vary according to diagnosis, and that the mean number of days was 26 among those with sick leave due to LBP [44]. Current Swedish recommendations for sick leave due to NSP and LBP also varies according to specific diagnosis [45]. Since the questions enquired about pain for the last 6 months, there is a risk of recall bias. Even though the STR is population based, it only contains twins born in Sweden and STAGE only contained young and middle-aged individuals, making the results generalizable only to these groups. Moreover, the response rate to STAGE was somewhat low and this may also have affected generalizability.

## Conclusion

To conclude, among those with low back and/or neck-shoulder pain, good health in terms of self-rated health and absence of mental or few pain sites, as well as good psychosocial working conditions seem to indicate a lower risk for work disability. For unemployment only absence of mental symptoms and high job control played a protective role. Furthermore, means to prevent work disability or unemployment that focus on increasing education may prove successful.

## Data Availability

The data that support the findings cannot be made publically available. According to the General Data Protection Regulation, the Swedish law SFS 2018:218, the Swedish Data Protection Act, the Swedish Ethical Review Act, and the Public Access to Information and Secrecy Act, these type of sensitive data can only be made available after legal review, for researchers who meet the criteria for access to this type of sensitive and confidential data. Readers may contact Associate Professor Pia Svedberg (pia.svedberg@ki.se) regarding the data.

## Abbreviations

CI: Confidence intervals
DSM-IV: Diagnostic and statistical manual of mental disorders, fourth edition
GAD: Generalized anxiety disorder
HR: Hazard ratios
LBP: Low back pain
LISA: Longitudinal Integration database for health insurance and labour market studies
MDD: Major depressive disorder
NSP: Neck and/or shoulder pain
OTC: Over the counter
SRH: Self-rated health
STAGE: Study of twin adults genes and environment
STODS: Swedish twin project of disability pension and sickness absence
STR: Swedish twin registry
